# Combining forces to target bacteria

**DOI:** 10.1111/j.1752-4571.2012.00255.x

**Published:** 2012-07-04

**Authors:** Dominik Refardt

**Affiliations:** Institute of Integrative BiologyETH Zürich, Switzerland

The enemy of my enemy is my friend. This proverb underlies the rationale to use viruses that specifically attack bacteria (that is bacteriophages, or phages for short) to treat bacterial infections. On paper, phages are the quintessential antibiotic. They can self-adjust their dose, they are species-specific in their target, they counter resistance through evolution, they have minimal side effects, and they can be mass-produced in a Petri dish (Loc-Carrillo and Abedon 2011). While reality is of course more complicated (see Thiel 2004 for a more sobering account), the idea of using phages to treat bacterial infections has gained momentum in the last decade owing to the global problem of resistance to chemical antibiotics (Levy and Marshall 2004). The goal is that phage therapy could complement or at times even replace chemical antibiotics. However, to attain this goal, the ecology and evolution of phage–bacteria interactions across multiple landscapes needs to be fleshed out. Two studies that make considerable headway into the complex world of phage–bacteria interactions are published in this issue of Evolutionary Applications (Escobar-Páramo et al. 2012; Zhang and Buckling 2012).

The parallels between the use of phages and chemical antibiotics to treat bacterial infections are obvious. For chemical antibiotics, an informed application of evolutionary and ecological concepts, both in theory and in practice, has proven valuable in understanding the determinants for efficient treatment and helps to provide guidelines for a sustainable drug use that minimizes the probability of resistance evolution. *In vitro* work has demonstrated that, although resistance mutations carry a fitness cost, counter-selection in the absence of an antibiotic can fail owing to compensatory adaptations that preclude a reversion to susceptibility (Andersson and Hughes 2010). High-throughput screens have uncovered interactions between antibiotics and have lead to the surprising insight that, although certain combinations are less efficient, they may hinder the evolution of multidrug resistance in the long term (Yeh et al. 2009). Theoretical work that employs population biological principles can be applied to suggest optimal treatment strategies or to evaluate the role of factors such as hospital size or bacterial mutation rate for the evolution of drug resistance (zur Wiesch et al. 2011).

However, much of our understanding of the ecology and evolution of antibiotic resistance arrived only after resistance had become extremely widespread. Phage therapy is not widely used at present in Western medicine, and by conducting the right studies now, we might be able to identify and implement treatment strategies that achieve long-term effectiveness with minimal spread of resistance – while doing some exciting fundamental evolutionary biology along the way. The range of topics related to phage therapy is vast and reads like the table of contents of an introductory textbook for ecology and evolution: host–parasite coevolution, resistance evolution, population extinction, predator-prey dynamics, virulence evolution, tri-trophic interactions, specialists versus generalists, to name a few.

Zhang & Buckling and Escobar-Páramo et al. address whether the combination of phages and antibiotics can enhance treatment success. Combination therapy, the use of more than one medication to treat a single disease, is a topic that is of practical relevance, as the combined use of different antibiotics is often the last resort against multidrug-resistant pathogens nowadays. Although it has the advantage of widening the range of bacteria that are targeted and, importantly, the promise of impeding resistance evolution in bacteria, the intake of several antibiotics can place a substantial burden on the patient, which can partially offset its benefits (Kett et al. 2011). Combining phage and antibiotic therapy is therefore an interesting alternative as experience with mixtures of phage strains suggests that they put minimal additional strain on the patient (Bruttin and Brüssow 2005; Kutter et al. 2010). Then again, different antimicrobials can interact in unforeseeable ways and the combined use of phages and antibiotics must therefore be evaluated carefully, which requires experiments like the two reported here.

Before discussing some of the findings, it is important to acknowledge that the experiments may seem a far stretch away from what is typically published under the title of phage therapy. Here, we are dealing with the most basic version of an infection, a bacterial monoculture in a test tube, which is passaged at regular intervals to new growth medium, and treated by adding phages or antibiotic, or both. The greater experimental control that is afforded by sacrificing the level of the patient allows more focus on specific factors, whose effect, both in isolation and in combination, can be measured much more accurately (Jessup et al. 2004). Because the individual bacterial populations (read: infections) are allowed to evolve before their response to treatments is assayed, the experiment provides insight into the evolutionary trajectories that are followed by pathogens given a particular treatment.

This reductionist approach yields encouraging results. First of all, the data are in line with basic expectations, which confirm that key assumptions are met and reasonable results can be obtained. Then, there are unexpected twists that remind us that phage therapy involves several evolving entities whose interactions can render the outcome very complex. Here, the two studies are particularly interesting because both were executed in a similar fashion. They use the same setup including the same bacterial species and bacteriophage, yet a different antibiotic, to address similar questions. Reading them side by side allows judging to what extent findings can be generalized, to what degree they must be explained as specific to the antibiotic used, and whether the inferences made are still valid when seen through the eyes of another researcher.

Both studies find that neither phage nor antibiotic are overly effective in eradicating a bacterial population, with the antibiotic doing better than the phage. Resistance against either was costly, but evidently offset by the advantages gained. By manipulating mutation rate and gene flow, the studies also point to the problem that changes in the evolutionary potential of the bacterial population can interfere with treatment success. However, treatment success changed dramatically when Zhang and Buckling combined antibiotic and phages. All but one of their 24 ‘infections’ were cured and in the single instance of treatment failure, bacteria paid a substantial fitness cost, which means that, given a functioning immune system, the infection might have been cleared as well. This is the good news. And now for the twists.

Escobar-Páramo and colleagues used combination therapy as well, this time with another antibiotic. Now, all bacterial populations survived. The phages went extinct in several replicate populations, which, challenged by the antibiotic alone, were able to persist. Treatment failure? It might depend on the mechanism responsible for the loss of the phages. First of all, loss might have been avoided if the phage had been applied repeatedly during the experiment (as it has been performed by Zhang and Buckling). Then, phages may have gone extinct exactly because the treatment was a success and the size of the bacterial population was intermittently so low, that phages were not able to persist anymore. Alternatively, and this is the more worrying scenario, the antibiotic may have interfered with the phages’ replication inside the bacterial cell (see Moss et al. 1969 for a similar example). If such antagonistic interactions occurred, it would mean that success of combination therapy depended critically on the type of antibiotic and phage used.

Another surprise came when phages and antibiotic were applied sequentially, with phages following the antibiotic. This caused the largest drop in bacterial population size of all treatments tested and suggests, as found by Zhang and Buckling, that costs for double resistance are substantial. Treatment success? When bacteria from the sequential treatment (which were all resistant to the antibiotic after the first part of the treatment) were assayed directly for antibiotic resistance at the end of the experiment, the majority was found to have lost antibiotic resistance. This even occurred when antibiotic treatment was continued throughout the experiment, both before and after phages were added. The fact that bacteria persist under these conditions indicate that they were resistant to the antibiotic under the experimental conditions. Closer examination suggested that they traded their genetic resistance with a mechanistic one, by acquiring the ability to form a biofilm. The buildup of such a structure during an infection can greatly complicate treatment, and the addition of the phage to the antibiotic may have moved us out of the frying pan into the fire.

This pair of studies is instructive in several ways. Most importantly, they provide evidence that combination therapy may indeed greatly enhance treatment success and that it should be followed up with experiments that study its use in an animal model. Additionally, these results must be interpreted very carefully with respect to treatment success or failure as clearing infections from test tubes may or may not reflect the recovery of a patient. Partially, this is due to the fact that treatment success is measured in different currencies in the two studies. While the extinction of a bacterial population is quite readily interpretable as clearing of an infection, declines in population size, competitive ability, or growth rate are more ambiguous. Resolving how such components of bacterial fitness contribute to treatment success is important, as it paves the way for future studies and how their results can be communicated.

What’s next from here? Ideally, the evolutionary dynamics of the phage–bacterium interaction should be examined in a patient or animal model. The immune system plays an important role in mediating any therapy, and bacterial virulence does not affect test tubes, but it does affect patients. Then, it is also important to make a clear distinction of the within- and between-patient levels. On the one hand, the aim is to cure the individual patient, and on the other hand, we are interested in the long-term evolutionary course of a pathogen population. There will be tensions between these two levels, and it is worthwhile to address them early on.
